# Climate drivers and winter constraints of dengue epidemics: a 10-year epidemiological perspective study in the Lao People’s Democratic Republic

**DOI:** 10.1186/s40249-026-01438-5

**Published:** 2026-05-01

**Authors:** Souphatsone Houatthongkham, Jae Hyun Kim, Bouaphanh Khamphaphongphane, Phonepadith Xangsayarath, Jong-Hun Kim, Sung Hye Kim

**Affiliations:** 1 Ministry of Health, Vientiane, Lao People’s Democratic Republic; 2 Department of Environmental Biology and Medical Parasitology, College of Medicine, Hanyang University, 222 Wangsimni-ro, Seoul, Republic of Korea; 3 Department of Social and Preventive Medicine, Sungkyunkwan University School of Medicine, Seobu-ro, Jangan-gu, Suwon-si, Gyeonggi-do 2066 Republic of Korea

**Keywords:** Dengue, Lao PDR, Threshold, Oceanic Niño index, Dipole mode index

## Abstract

**Background:**

Dengue fever is hyperendemic in the Lao People’s Democratic Republic (PDR), where transmission is driven by *Aedes* mosquitoes and influenced by large-scale climatic phenomena, including the El Niño-Southern Oscillation (ENSO) and the Indian Ocean Dipole (IOD). As a landlocked nation, the Lao PDR experiences sharper winter temperature declines than coastal regions, which may impose a seasonal “bottleneck” on vector survival and dengue transmission. This study examined whether winter minimum temperatures act as a seasonal transmission bottleneck, alongside the Oceanic Niño Index (ONI) and the Dipole Mode Index (DMI), during 2014–2023.

**Methods:**

Monthly dengue case counts reported to the National Center for Laboratory and Epidemiology, Ministry of Health, Lao PDR, from January 2014 to December 2023 were analyzed using region-specific quasi-Poisson distributed lag nonlinear models. Models incorporated 3-month-lagged ONI/DMI cross-basis functions, winter minimum temperature hinges, long-term trends, and seasonality, with population as an offset. Region-specific estimates were pooled using multivariate meta-analysis to generate best linear unbiased predictions (BLUPs). Optimal lag structures and temperature thresholds were selected by minimizing the quasi-Akaike information criterion and residual sum of squares.

**Results:**

A total of 134,093 dengue cases were reported, with substantial regional heterogeneity. The Capital Region had the highest burden (40,672 cases; annual incidence 35.4 per 100,000), followed by the Southern Mountains and Tropical Rainforests region (20,176 cases; 23.3 per 100,000). Annual incidence in each region appeared constrained by region-specific winter minimum temperature thresholds. Pooled BLUPs analyses adjusted for covariates revealed monotonic cumulative relative risk increases with ONI [RR = 2.83 at ONI = 2.0; 95% confidence interval (*CI*): 1.46–5.49) and decreases with DMI (RR = 0.37 at DMI = 1.5; 95% *CI:* 0.24–0.59).

**Conclusions:**

Winter cold functions as a primary bottleneck for dengue transmission in the Lao PDR, with ENSO amplifying and IOD suppressing outbreak risk. These findings support the development of climate-integrated, region-specific early warning systems. Incorporating 3-month-lagged climate indices may enhance public health preparedness for future dengue outbreaks.

**Graphical Abstract:**

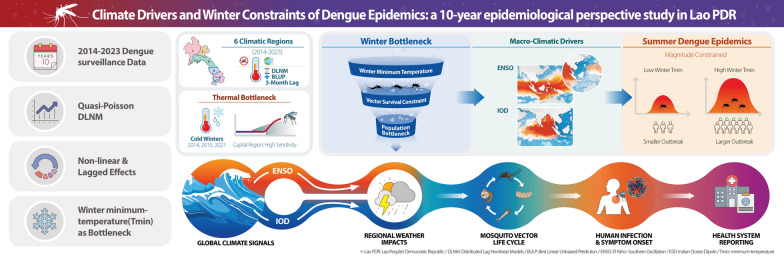

**Supplementary Information:**

The online version contains supplementary material available at 10.1186/s40249-026-01438-5.

## Background

Dengue has emerged as a major global public health concern, with incidence increasing markedly over recent decades. In 2023 alone, over 6.5 million cases and more than 7,300 deaths were reported worldwide [1]. The primary vectors, *Aedes aegypti and Ae. albopictus*, thrive in urban environments, where rapid urbanization—characterized by high population density and inadequate infrastructure—has intensified viral transmission [2–5]. Beyond urban growth, climate change is increasingly recognized as reshaping the seasonal suitability and geographic distribution of *Aedes*-borne virus transmission. Temperature-dependent “windows” of transmission potential can expand or shift as mean temperatures and extremes change over time [6].

Transmission dynamics are strongly influenced by climatic factors, particularly temperature and rainfall, which affect mosquito breeding, survival, and the extrinsic incubation period [7]. Higher temperatures typically accelerate vector development and viral replication [8]; however, temperature-dengue relationships are frequently nonlinear, reflecting trade-offs between faster viral replication and reduced adult survival at very high temperatures [9]. Rainfall effects are similarly complex; precipitation can create essential breeding sites, whereas excessive rainfall may flush out larvae [10]. Additional weather dimensions—including relative humidity, which influences adult survival and host-seeking activity, and wind patterns, which can affect mosquito dispersal—have also been associated with dengue incidence, often with time lags [9, 11]. Importantly, natural environments rarely experience constant temperatures; diurnal temperature range can modify mosquito survival and vector competence, such that identical mean temperature may yield different transmission potentials depending on daily fluctuation patterns [12]. These nonlinear and lagged relationships support the use of models that explicitly incorporate delayed effects across several weeks, consistent with mosquito life cycles and incubation periods [13].

Large-scale climate phenomena, such as the El Niño-Southern Oscillation (ENSO) and the Indian Ocean Dipole (IOD), further influence regional weather patterns through ocean–atmosphere teleconnections [14–16]. These phenomena are monitored using standardized indices, including the Oceanic Niño Index (ONI) for ENSO, and the Dipole Mode Index (DMI) for the IOD [17, 18]. Because variability in the Pacific and Indian Oceans can precede local anomalies in rainfall and temperature, these indices may provide longer lead times for dengue early warning than local weather variables alone. Prior studies have shown that the ONI influences dengue risk indirectly through its effects on local rainfall and temperature, supporting the use of ENSO-aware frameworks for outbreak preparedness [13]. More recently, global analyses have emphasized that Indian Ocean sea-surface temperature anomalies may help anticipate longer-term dengue trends, complementing ENSO-focused approaches [19]. Collectively, these findings suggest that large-scale climate signals contribute to interannual variation in dengue burden, although their effects likely vary by local ecology and baseline seasonality.

Despite strong evidence linking climate to dengue, few studies have explored the specific effects of ENSO and the IOD on dengue incidence within the Lao People’s Democratic Republic (PDR). A critical ecological feature of the country is its winter climate, which may function as a seasonal “bottleneck.” In regions where winter minimum temperatures fall below specific thermal thresholds, the survival of overwintering mosquito populations is restricted [20–22]. Experimental and field evidence from other settings indicates that cold-season constraints can substantially limit persistence across life stages, although microclimatic refuges—such as water storage containers that remain warmer than ambient air—may partially offset these effects. These findings underscore the importance of minimum temperatures and shelter availability for seasonal vector carryover [23, 24]. Consequently, in climates with cooler winters, the magnitude of subsequent dengue transmission may depend not only on warm-season conditions and ocean-driven anomalies, but also on whether the vector population can persist through the coldest period. Nevertheless, existing research rarely addresses whether and how winter climate acts as a “bottleneck” that shapes the initial conditions of the following transmission season.

Within the Lao PDR, dengue exhibits marked seasonality and substantial spatial heterogeneity. Recent national and subnational analyses consistently report annual peaks during the rainy season (typically from June to September), alongside pronounced geographic variation across districts and provinces [4, 25, 26]. A nationwide time-series study found that dengue risk generally increased with rising temperatures and moderate rainfall but decreased under extreme rainfall, highlighting nonlinear precipitation effects and regional heterogeneity in weather-dengue associations [4]. District-level spatiotemporal modeling has further identified high-risk clusters and associations with temperature and remotely sensed environmental indicators, demonstrating that climate signals act within broader landscape contexts (e.g., vegetation and land-surface conditions) [25]. Complementing these climate-focused findings, virologic surveillance indicates that all four dengue virus serotypes circulate in Lao PDR and that shifts in dominant serotypes have accompanied major outbreaks, reinforcing the need to interpret climate-incidence relationships within a dynamic immuno-epidemiological context [27, 28].

This study addressed these knowledge gaps by examining the relationship between dengue incidence and climate variability in Lao PDR from 2014 to 2023. Specifically, it aimed to quantify the distinct influences of Pacific and Indian Ocean climate systems while accounting for the restrictive role of localized seasonal temperature constraints.

## Methods

### Study area and regional classification

Lao PDR is a landlocked nation in Southeast Asia where dengue fever is hyperendemic, characterized by the continuous circulation of all four virus serotypes [29]. The disease imposes a substantial public health burden. Notably, a major outbreak in 2019 resulted in 39,901 cases and 76 deaths, surpassing the per capita incidence observed in several neighboring countries [30].

**Fig. 1:**
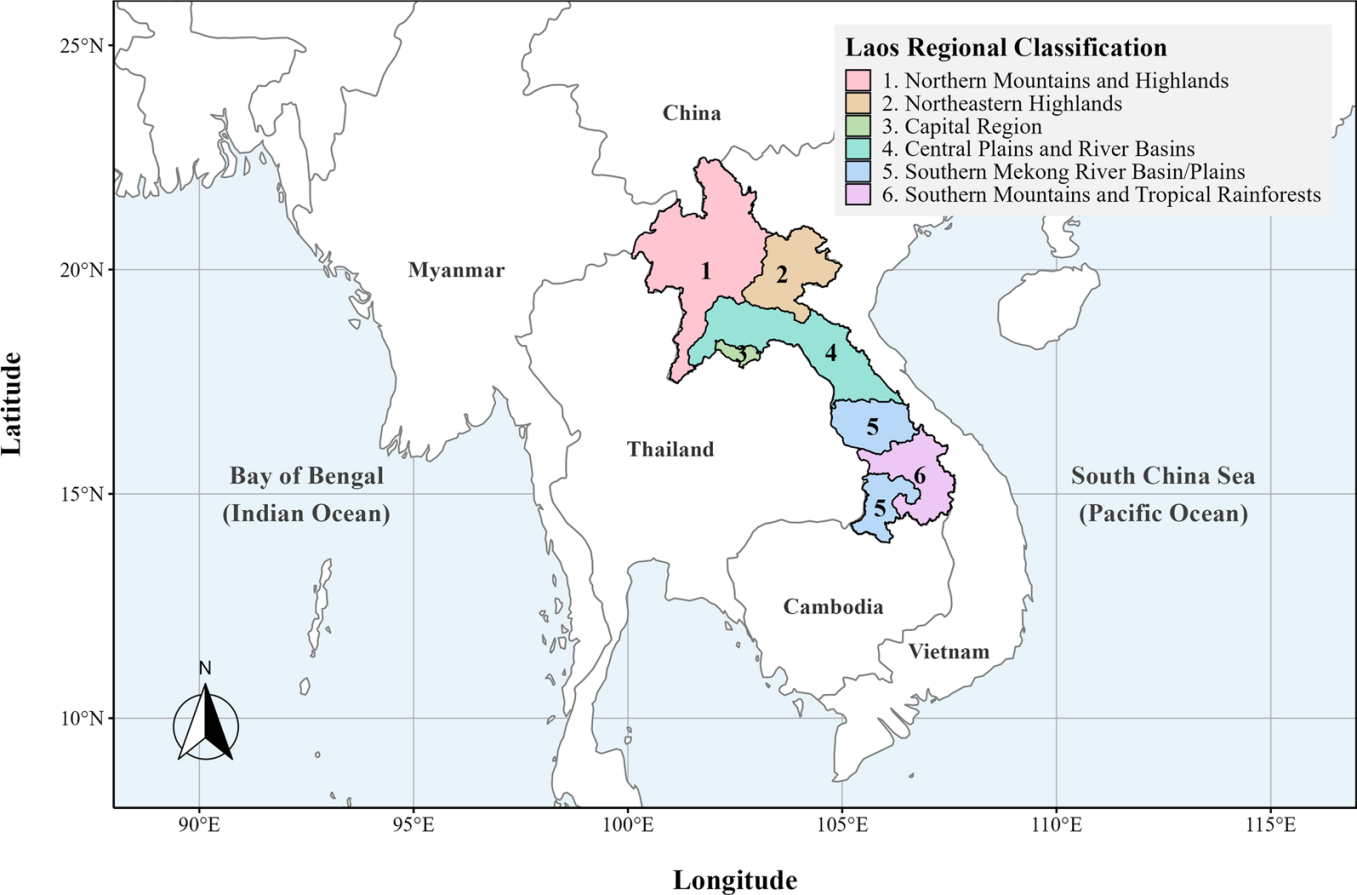
Regional classification of the Lao People’s Democratic Republic based on topographical and climatic characteristics

Transmission is primarily driven by the mosquito vectors *Ae. aegypti* and *Ae. albopictus*, which thrive in urban centers such as Vientiane Capital. Rapid urbanization and high population density in these areas facilitate efficient viral spread [4, 5]. To account for the country's heterogeneous topography and environmental conditions, the study area encompasses 18 administrative units grouped into six climatic and ecological regions (Fig. 1; Supplementary Table S1): (ⅰ) Northern Mountains and Highlands, (ⅱ) Northeastern Highlands, (ⅲ) Capital Region, (ⅳ) Central Plains and River Basins, (ⅴ) Southern Mekong River Basin/Plains, and (ⅵ) Southern Mountains and Tropical Rainforests.

### Conceptual framework: the climate-dengue transmission chain

Dengue transmission is a multifaceted process linking large-scale climate variability to local mosquito ecology and human health systems. This study applies a conceptual framework (Supplementary Figure S1) to evaluate how oceanic climate phenomena influence regional disease dynamics through a sequence of biological and environmental pathways.

### Research integration

To quantitatively assess these linkages, we assembled an integrated dataset representing multiple stages of the transmission pathway. The dataset combines 10 years of nationally reported dengue case data (2014–2023), local meteorological variables, and global climate indices (ONI and DMI). We applied a Distributed Lag Nonlinear Model (DLNM) [31, 32] to disentangle the independent effects of the Pacific and Indian Ocean systems while accounting for biologically plausible delays inherent in dengue epidemiology.

### Data collection

Monthly dengue incidence data for all 17 provinces and Vientiane Capital from January 2014 to December 2023 were acquired from the National Center for Laboratory and Epidemiology, Ministry of Health, Lao PDR (Supplementary Table S2). To facilitate the calculation of incidence rates, provincial-level population statistics for the corresponding period were obtained from the Lao Statistics Bureau. Local meteorological variables, including temperature and precipitation, were provided by the Department of Meteorology and Hydrology, Ministry of Natural Resources and Environment, Lao PDR. Regional monthly meteorological summaries were also obtained from the Lao Statistics Bureau.

Large-scale ocean climate variability was quantified using monthly indices for the ENSO and the IOD obtained from the Asia-Pacific Economic Cooperation Climate Center. The ONI, defined as a 3-month running mean of sea surface temperature (SST) anomalies in the Niño 3.4 region (5°N–5°S, 120°–170°W), served as the primary ENSO metric. El Niño conditions were defined as ONI anomalies ≥ + 0.5 °C persisting for at least five consecutive overlapping periods**,** whereas La Niña conditions were defined as anomalies ≤ − 0.5 °C. Any periods where the ONI anomalies remained between these thresholds were classified as ENSO-neutral.

Similarly**,** the DMI was utilized to characterize the IOD by measuring the SST anomaly gradient between the Western Tropical Indian Ocean (50°–70°E, 10°S–10°N) and the Southeastern Tropical Indian Ocean (90°–110°E, 10°–0°S). Based on the National Oceanic and Atmospheric Administration’s Extended Reconstructed Sea Surface Temperature version 5 dataset, a positive IOD phase was defined as DMI anomalies > + 0.5**,** indicating warmer-than-average waters in the western pole. Conversely, a negative IOD phase was defined as anomalies < − 0.5, while values between these thresholds were categorized as neutral, indicating no significant dipole pattern.

### Statistical analysis

To account for the overdispersion in dengue case counts, we fitted a quasi-Poisson DLNM for each region. Monthly case counts were modeled as a function of a winter minimum-temperature hinge term, large-scale climate indices (ONI and DMI), and a long-term temporal trend, with seasonality controlled using harmonic terms. Population size was included as an offset to model incidence rates. Cross-basis functions for ONI and DMI were constructed to capture potential lagged effects of up to 8 months, accounting for delays in mosquito population dynamics and disease transmission. Final lag structures were refined by minimizing the cumulative Quasi-Akaike Information Criterion (QAIC) across all regions, reflecting the overall goodness-of-fit of the lag-response relationship. For each index, the exposure-response relationship was specified as linear, while lagged effects were modeled using a discrete integer lag structure.

The winter minimum temperature threshold associated with annual dengue incidence was identified using hinge regression applied to annualized data. Annual dengue incidence was defined as the total number of cases per 100,000 population based on mid-year estimates. Winter minimum temperature (*TminDJF*) was defined as the lowest recorded temperature between December and February. Models were adjusted for long-term trends, mean summer temperature (June–September), and cumulative summer precipitation, reflecting the primary transmission season. For each region, we fitted a series of candidate segmented regression models in which the effect of *TminDJF* was modeled using a hinge function defined as follows:

$${\mathrm{Incidence}}\,{ = }\,\beta_{0} \, + \,\beta_{1} \,TminDJF\, + \,\beta_{2} \,\left( {TminDJF\, - \,\tau } \right)\, + \,\gamma X\, + \, \in$$

Here, τ represents the candidate threshold, and X denotes a set of covariates used to control for long-term trends, mean summer temperature (June–September), and cumulative summer precipitation. To focus on the hypothesized “winter bottleneck” mechanism and exclude questionably high values, candidate thresholds were restricted to the lower 50th percentile of the *TminDJF* distribution. Each model was fitted using ordinary least squares regression, and the optimal threshold (τ) was selected by minimizing the residual sum of squares.

The effects of ONI, DMI, and winter minimum temperature were estimated individually for each region. Because data volume and estimate precision varied geographically, regional results were pooled using a multivariate meta-analytic approach. This method accounts for both within-region uncertainty and between-region heterogeneity. The resulting national association corresponds to a best linear unbiased prediction (BLUP), which allows for genuine regional differences while providing a stable and interpretable summary of climate effects on national dengue epidemics [31].

For each region *r* and month *t*, the expected number of dengue cases ${\mu }_{r,t}$ was estimated using the following model:

$${Cases}_{r, t} \sim quasi-Poisson\left({\mu }_{r,t}\right)$$

$$\mathrm{log}\left({\mu }_{r,t}\right)={\alpha }_{r}+{\beta }_{1}\hspace{0.17em}{H\left(\tau \right)}_{r,t}+{f}_{ONI}\left({ONI}_{r,t}\right)+{f}_{DMI}\left({DMI}_{r,t}\right)+{\beta }_{2}\hspace{0.17em}{Year}_{t}+{\beta }_{3}sin\left(\frac{2\pi \hspace{0.17em}{Month}_{t}}{12}\right)+{\beta }_{4}cos\left(\frac{2\pi \hspace{0.17em}{Month}_{t}}{12}\right)+\mathrm{log}\left({Population}_{r,t}\right)$$

In this model, ${\alpha }_{r}$ is the region-specific intercept, and H(τ)_r,t_ represents the hinge function for region r at time t, defined as the positive difference between the winter minimum temperature and the region-specific thermal threshold. The functions ${f}_{ONI}(\bullet )$ and ${f}_{DMI}(\bullet )$ denote distributed lag cross-basis terms for the Pacific and Indian Ocean indices, respectively. The Year_t_ term represents long-term temporal trends, and the sine and cosine terms account for seasonal periodicity.

To evaluate robustness and characterize temporal relationships between climate indices and dengue incidence, we conducted sensitivity analyses by varying time lags from 0 to 8 months. For each interval, quasi-Poisson regression models were fitted independently for the six study regions. Model selection was guided by QAIC, which is appropriate for over-dispersed count data. For each candidate lag structure, QAIC values were calculated by region and summed to obtain a cumulative QAIC, representing the overall goodness-of-fit of the lag–response relationship. The lag specification minimizing this cumulative QAIC was selected as the optimal and most parsimonious model. Model performance was further evaluated using mean adjusted pseudo-R^2^ across regions to confirm the stability of the selected lag structure.

All statistical analyses were performed using R software (version 4.4.3; R Foundation for Statistical Computing, Vienna, Austria). DLNMs were implemented using the *dlnm* package to characterize potentially delayed climate effects on dengue incidence. Multivariate meta-analysis for pooling regional estimates was performed using the *mvmeta* package. Hinge regression for threshold identification was implemented using base R functions.

### Ethical considerations

Ethical approval for secondary data analysis was obtained from the Institutional Review Board (IRB) of Sungkyunkwan University (IRB file no. 2025-02-010).

## Results

### Dengue incidence and climate patterns across regions

**Fig. 2:**
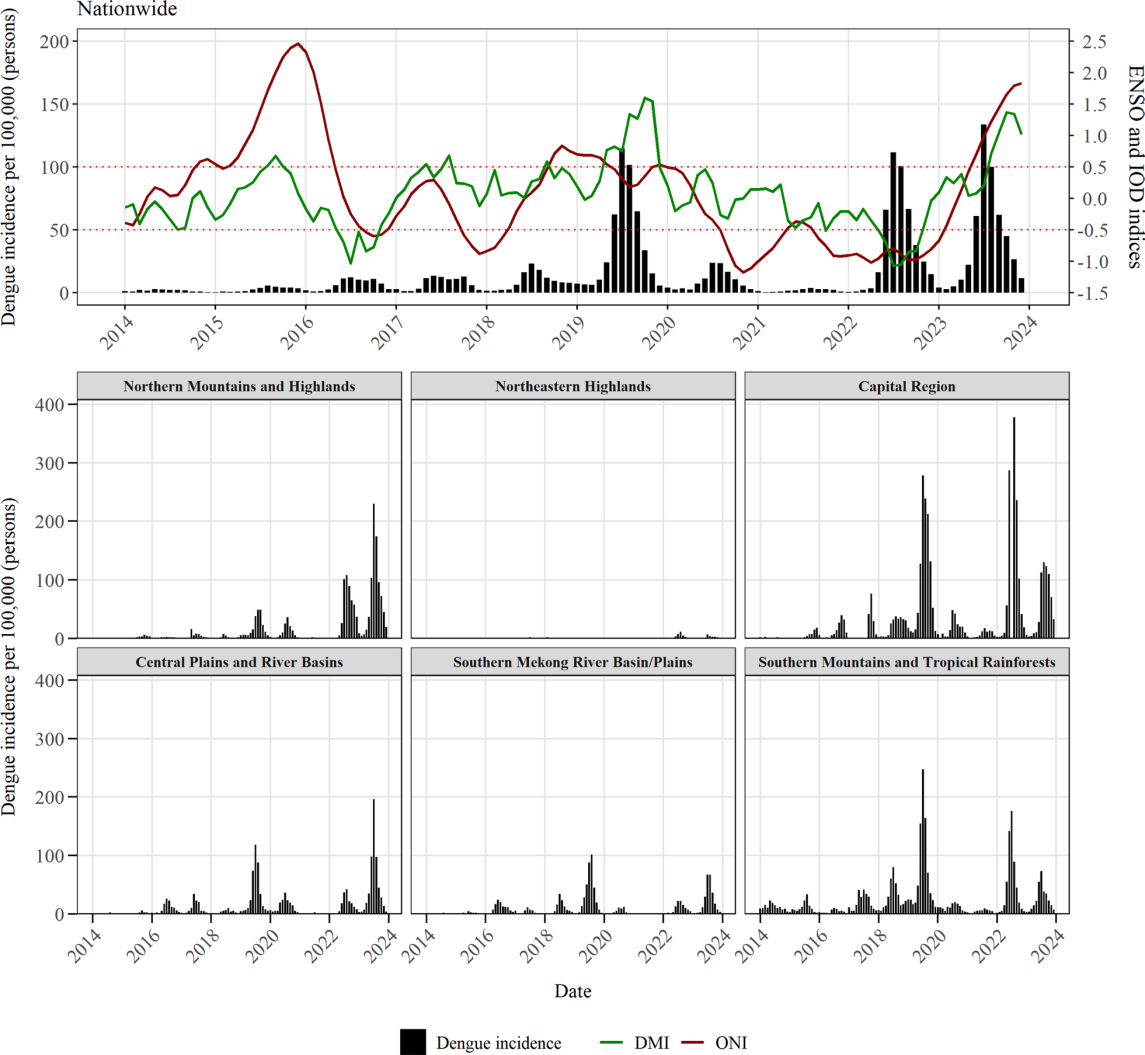
Regional and nationwide temporal variability in dengue incidence in relation to ONI and DMI. *ENSO* El Niño–Southern Oscillation, *IOD* Indian Ocean Dipole, *DMI* Dipole Mode Index, *ONI* Oceanic Niño Index

Between 2014 and 2023, a total of 134,093 dengue cases were reported across the six climatic regions of Lao PDR, revealing significant regional heterogeneity in both disease burden and environmental conditions (Table 1). Temporal patterns were broadly consistent with major national peaks observed in 2016–2017, 2019, 2020, 2022, and 2023; however, outbreak magnitude varied significantly by region, with particularly large nationwide epidemics in 2019, 2022, and 2023 (Fig. 2). The Capital Region experienced the most intense and explosive outbreaks, with an incidence exceeding approximately 400 cases per 100,000 population in July 2022, representing the highest regional peak during the study period. The Northern Mountains and Highlands and the Central Plains and River Basins experienced major outbreaks in July 2023, with peak incidences of approximately 230 per 100,000 and 200 per 100,000, respectively. In contrast, during the 2019 epidemic, the Southern Mountains and Tropical Rainforests exhibited a pronounced peak of approximately 250 cases per 100,000 population in July 2019, whereas the Southern Mekong River Basin/Plains exhibited a more moderate peak of approximately 100 cases per 100,000 population in August 2019.

**Table 1:** Descriptive statistics for dengue cases, population, and climate variables across six climatic regions in Lao PDR, 2014–2023

Regional classification	Population (10-year average)	Total dengue cases	Annual incidence over 10 years (per 100,000)	Mean temperature (°C, SD)	Median monthly precipitation (mm, IQR)
Northern mountains and highlands	1,796,700	33,180	14.8	24.3 (3.0)	87.6 (135.3)
Northeastern highlands	576,600	400	0.6	21.9 (3.4)	93.4 (141.3)
Capital region	914,900	40,672	35.4	27.3 (2.2)	73.6 (193.3)
Central plains and river basins	1,289,000	20,753	13.1	27.3 (2.2)	113.2 (335.6)
Southern Mekong River basin/plains	1,780,400	18,912	8.7	27.3 (2.2)	76.0 (255.0)
Southern mountains and tropical rainforests	710,100	20,176	23.3	27.8 (1.8)	90.4 (263.8)

*SD* Standard deviation, *IQR* Interquartile range, *PDR* People’s Democratic Republic

Urban centers, particularly Vientiane Capital, consistently recorded the highest per capita incidence (35.4 cases per 100,000) and total case counts (40,672 cases) over the decade (Table 1; Supplementary Figure S2). Dengue incidence was highly heterogeneous across regions, with the second highest average incidence (23.3 cases per 100,000) observed in the Southern Mountains and Tropical Rainforests, whereas the Northeastern Highlands had the lowest incidence (0.6 cases per 100,000), with only 400 cases reported during 2014–2023. Even regions with lower cumulative counts, such as the Southern Mekong River Basin/Plains (8.7 cases per 100,000; 18,912 total cases), contributed substantially to the national burden, accounting for over 14% of all reported cases.

Marked regional differences were observed in average temperature and precipitation across the six climatic regions (Table 1). The northern mountainous regions exhibited relatively low mean annual temperatures (< 25 °C) and greater seasonal variation between winter (< 20 °C) and summer (25–26 °C) temperatures compared with other regions, where winter temperatures averaged 24–25 °C and summer temperatures were approximately 28 °C (Supplementary Figure S3). In contrast, mean annual temperatures in the remaining regions were similar, at approximately 27.5 °C. Precipitation patterns also varied substantially by region, showing clear dry and wet seasons (Supplementary Figure S4). The Capital Region experienced the lowest median monthly precipitation (73.6 mm), whereas the Central Plains and River Basins (113.2 mm), Southern Mekong River Basin/Plains (76.0 mm), and Southern Mountains and Tropical Rainforests (90.4 mm) experienced higher precipitation levels (Table 1).

### Model selection and lag determination

The primary modeling framework was selected by balancing statistical performance and operational applicability. Sensitivity analysis indicated that a 0-month lag provided the best statistical fit (total QAIC = 604.76); however, a 3-month lag model was selected for the final analysis (QAIC = 666.24) (Supplementary Table S3). This lag structure was selected to enhance operational feasibility for public health early warning systems by accounting for the inherent latency in the verification and release of climate indices (Supplementary Figure S5). Under the selected 3-month lag model, the mean adjusted pseudo-*R*^2^ across the regions was 0.78 (range: 0.59–0.85), indicating a strong explanatory power for the observed fluctuations in dengue incidence.

### Winter minimum temperature as a seasonal bottleneck

**Fig. 3:**
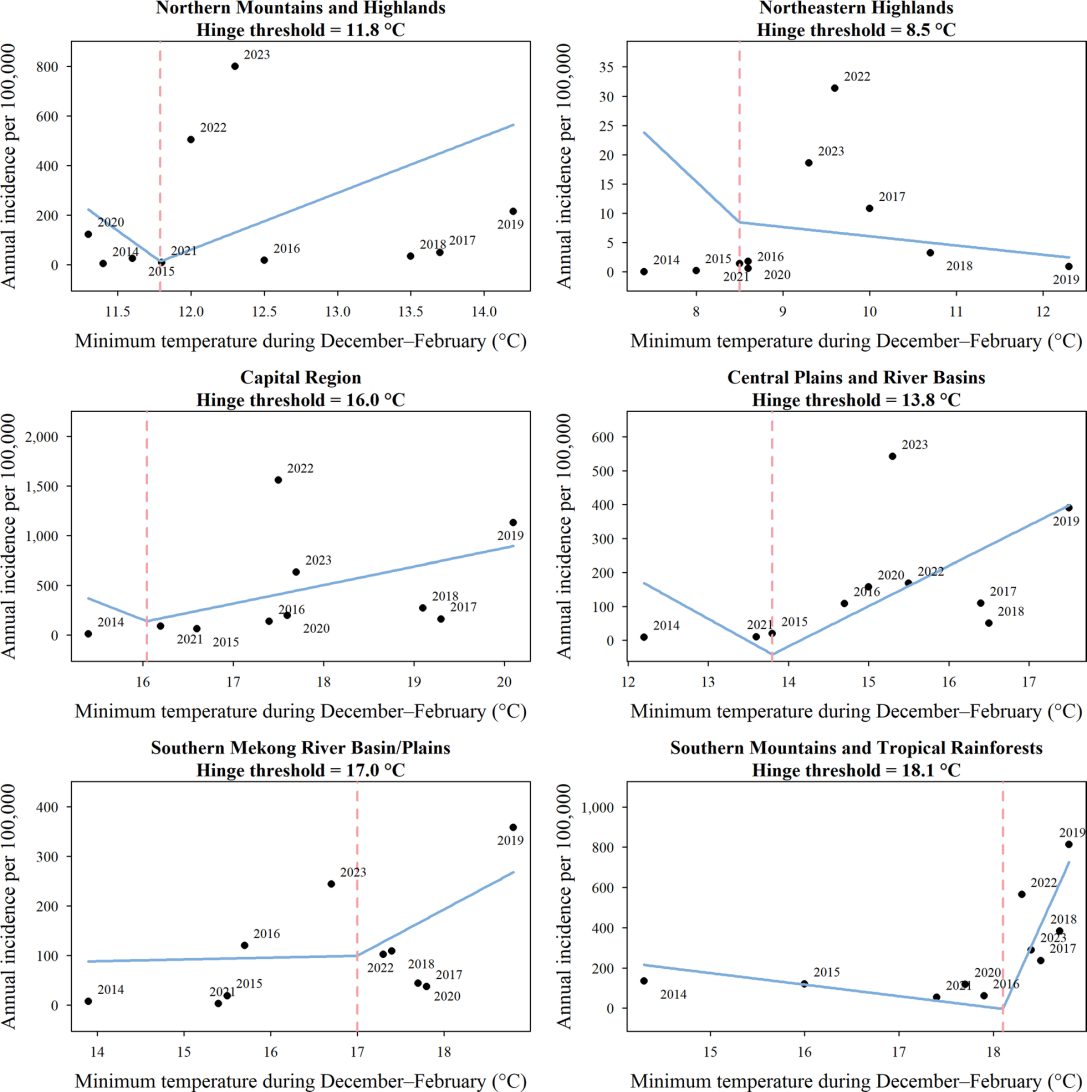
Region-specific winter minimum temperature thresholds associated with annual dengue incidence

**Fig. 4:**
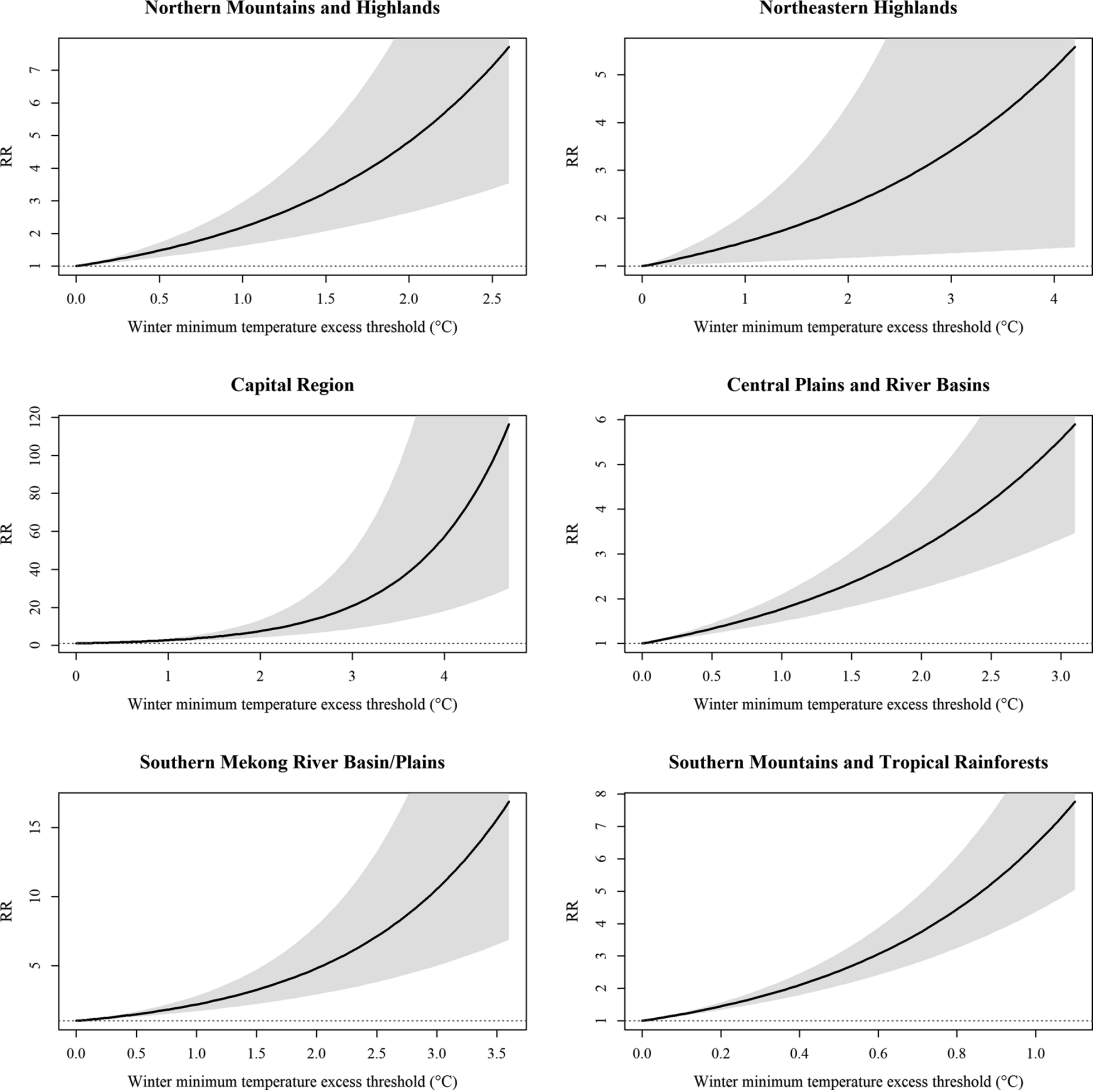
Region-specific relative risks of dengue incidence associated with winter minimum temperature exceedance above threshold. *RR* Relative Risk

Using region-specific hinge regression, this study identified a critical winter minimum temperature threshold below which annual dengue incidence remained consistently low or exhibited only a weak variation (Fig. 3). In the Southern Mountains and Tropical Rainforests, this threshold was estimated at 18.1 °C. Years with winter temperatures below this threshold averaged 102 cases per 100,000 population, whereas years exceeding it averaged 457 cases per 100,000 population. At the national level, unusually cold winters—notably in 2014, 2015, and 2021—coincided with exceptionally low annual dengue incidence. This pattern supports the hypothesis that winter cold acts as a seasonal bottleneck in dengue transmission. Once winter temperatures surpassed these critical hinge points, epidemiological responses became markedly heterogeneous. The Capital Region exhibited the greatest sensitivity to temperature exceedances (approximately 5 °C above the threshold) and the largest increase in relative risk (Fig. 4). This pattern may help explain why dengue incidence did not surge in the capital during 2015–2016 despite a strong El Niño event, as winter temperatures remained below the identified ecological threshold required for widespread transmission during that period.

### Associations between climate indices and regional dengue risk

**Fig. 5:**
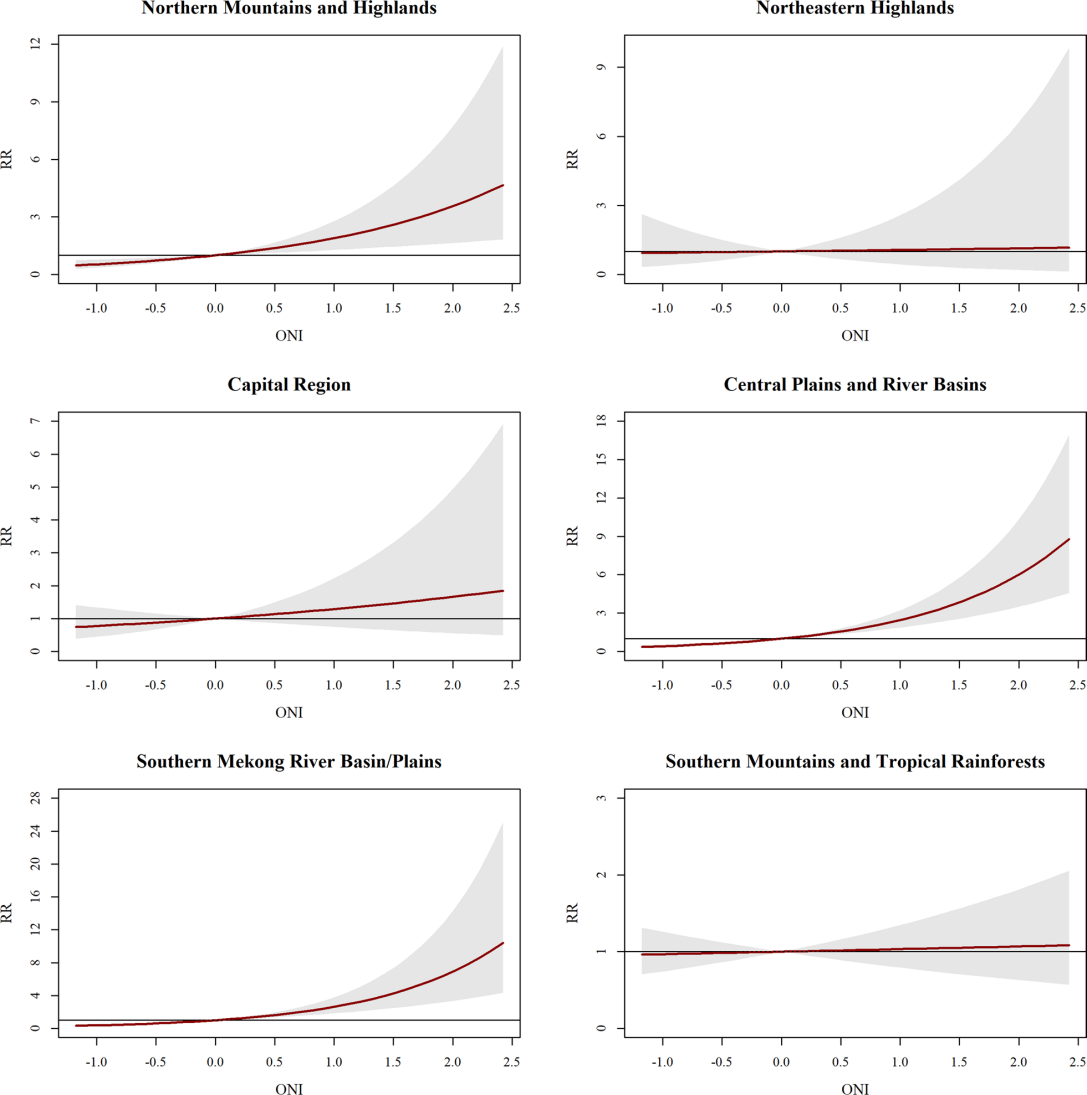
Cumulative relative risk of dengue incidence associated with ONI across Lao People’s Democratic Republic regions. *RR* Relative Risk, *ONI* Oceanic Niño Index

**Fig. 6:**
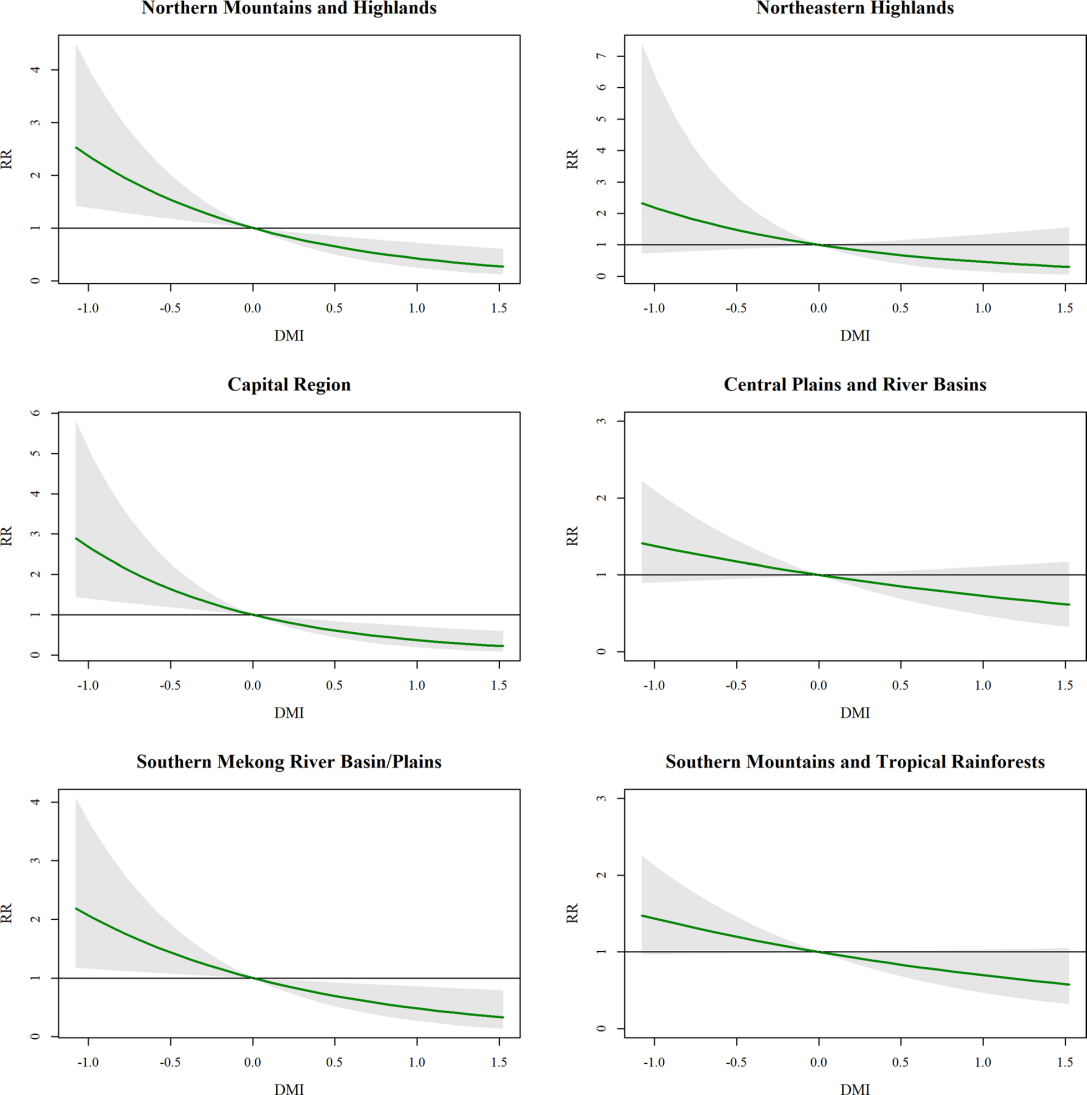
Cumulative relative risk of dengue incidence associated with DMI across Lao People’s Democratic Republic regions. *RR* Relative Risk, *DMI* Dipole Mode Index

Elevated ONI values were strongly associated with increased dengue risk (Fig. 5; Supplementary Table S4). In the Northern Mountains, Central Plains, and Southern Mekong Basin, cumulative relative risks (CRR) increased monotonically with rising ONI. Specifically, an ONI of 2.0 was linked to a CRR of 6.02 in the Central Plains and 6.93 in the Southern Mekong Basin. Conversely, the DMI demonstrated a negative protective association across most regions (Fig. 6; Supplementary Table S5). In the Northern Mountains and Highlands, an increase in DMI to 1.5 corresponded to a significantly reduced CRR of 0.28, while the Capital Region showed a reduction to 0.23 at the same DMI level.

### Pooled national-level trends

**Fig. 7:**
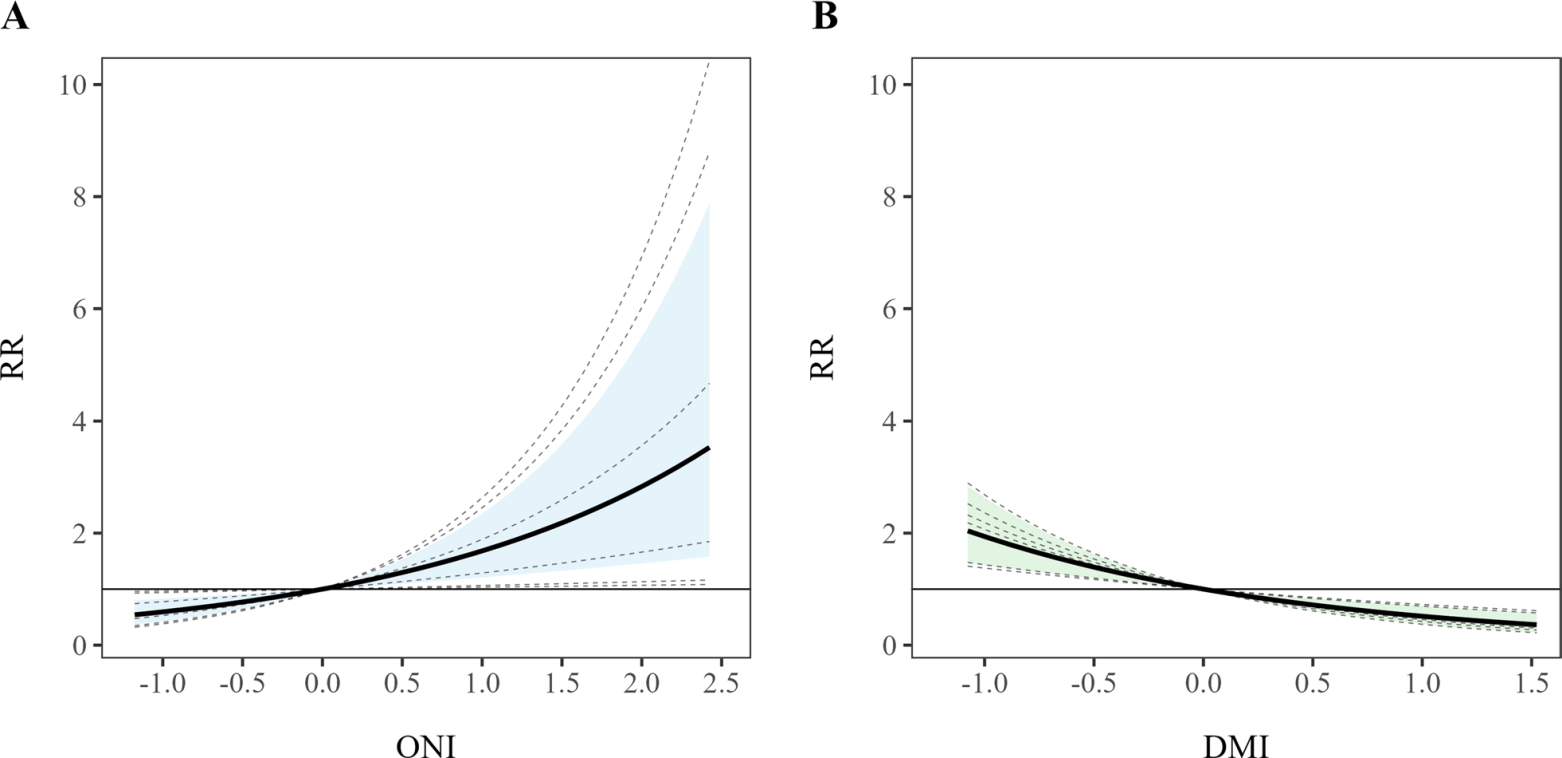
**A** Pooled national-level cumulative relative risk for the Oceanic Niño Index, Lao People’s Democratic Republic. *RR* Relative Risk, *ONI* Oceanic Niño Index. **B** Pooled national-level cumulative relative risk for the Dipole Mode Index, Lao People’s Democratic Republic. *RR* Relative Risk, *DMI* Dipole Mode Index

Pooled estimates using BLUP summarized the overall pattern of association between oceanic climate drivers and dengue incidence across regions (Table 2; Fig. 7). The pooled CRR for ONI increased monotonically with rising ONI values, reaching 2.83 [95% confidence interval (*CI*): 1.46–5.49] at an ONI of 2.0. In contrast, the pooled exposure–response curve for the DMI showed a monotonic decrease, with a DMI value of 1.5 associated with a reduced relative risk of 0.37 (95% *CI:* 0.24–0.59). Overall, these BULP-based pooled curves suggest that positive ENSO phases are associated with elevated dengue risk in Lao PDR, whereas positive IOD phases are associated with substantial attenuation of dengue transmission.

**Table 2:** Best Linear Unbiased Predictions of cumulative relative risk for dengue incidence in Lao People’s Democratic Republic associated with the Oceanic Niño Index and Dipole Mode Index, derived from multivariate meta-analysis of region-specific quasi-Poisson distributed lag nonlinear models, 2014–2023

Climate indices	Value						
	–1.0	–0.5	0	0.5	1.0	1.5	2.0
ONI	0.59 (0.43–0.83)	0.77 (0.65–0.91)	1.00 (0.99–1.01)	1.30 (1.10–1.53)	1.68 (1.21–2.35)	2.18 (1.33–3.59)	2.83 (1.46–5.49)
DMI	1.94 (1.43–2.63)	1.39 (1.20–1.62)	1.00 (0.99–1.01)	0.72 (0.62–0.84)	0.52 (0.38–0.70)	0.37 (0.24–0.59)	–

*ONI* Oceanic Niño Index, *DMI* Dipole Mode Index

## Discussion

Building on the observed spatiotemporal patterns of dengue in Lao PDR, this study shows that interannual dengue incidence is shaped by a winter minimum-temperature “bottleneck effect” that conditions how strongly large-scale ocean-atmosphere variability (ENSO and the IOD) translates into outbreak risk. Using DLNMs for 2014–2023, while accounting for long-term trends and seasonality, we found a consistent direction of association across regions: warmer winter minima were associated with higher subsequent dengue risk; positive ENSO conditions (higher ONI) were associated with increased risk; and positive IOD conditions (higher DMI) tended to be associated with reduced risk.

These results support a hierarchical interpretation of climate influence. When winter minimum temperatures fall below region-specific thermal thresholds, vector persistence into the subsequent season is likely constrained, thereby limiting the scale of subsequent transmission even if broader climate signals become favorable later in the year [33–35]. Once this local constraint is relaxed, the ONI and DMI appear to modulate risk through their influence on seasonal transitions and baseline environmental suitability. Notable regional heterogeneity was observed: the Capital Region showed greater sensitivity to modest winter warming than to the broader ONI variability, suggesting that dense urban settings may amplify local thermal effects while partially decoupling transmission from remote climate teleconnections [36].

For public health application, a 3-month lag was selected as a pragmatic compromise between biological plausibility and operational feasibility, given the reporting structure of ONI/DMI and the time required for mosquito population growth and viral incubation. Collectively, these findings indicate that climate-informed early warning in Lao PDR should be region-specific and staged: (i) assess whether winter temperatures exceed local thresholds that permit early-season vector carryover, and (ii) incorporate Pacific and Indian Ocean climate indices to anticipate subsequent amplification or suppression of risk. Key limitations remain, including underreporting in passive surveillance systems and the omission of entomological, socioeconomic, mobility, and immunological factors (including serotype dynamics). Nevertheless, integrating winter thermal thresholds with ONI/DMI provides a clearer mechanistic basis for targeted preparedness than models relying on teleconnections alone.

Despite these limitations, a principal strength of this study lies in the novel integration of winter thermal thresholds with large-scale climate indices. By identifying the “bottleneck effect,” this study provides a more comprehensive framework for understanding how local seasonal constraints and remote climate drivers jointly shape dengue risk. These findings provide empirical support for developing climate-informed, region-specific early warning strategies to enhance public health preparedness in Lao PDR.

## Conclusions

Dengue risk in Lao PDR reflects a layered climate process in which winter minimum temperatures function as a primary ecological gatekeeper, while ENSO/IOD variability modulates risk once this constraint is relaxed. A 3-month lag provides a feasible lead time for prediction using ONI and DMI, supporting the development of operational, region-specific dengue early warning strategies that explicitly incorporate winter thermal thresholds.

## Supplementary Information

Supplementary Material 1.

## Data Availability

The dengue surveillance data supporting the findings of this study are available from the National Center for Laboratory and Epidemiology, Ministry of Health, Lao PDR, but access is subject to restrictions. Data are, however, available from the authors upon reasonable request and with permission from the Ministry of Health.

## Abbreviations

BLUP: Best linear unbiased prediction
*CI*: Confidence interval
CRR: Cumulative relative risk
DLNMs: Distributed lag nonlinear models (also referred to as quasi-Poisson distributed lag nonlinear model).
DMI: Dipole mode index
ENSO: El Niño-Southern Oscillation
IOD: Indian Ocean Dipole
IQR: Interquartile range
IRB: Institutional Review Board
Lao PDR: Lao People's Democratic Republic
ONI: Oceanic Niño Index
QAIC: Quasi-Akaike Information Criterion
RR: Relative risk
SD: Standard deviation
SST: Sea surface temperature

## References

[1] WHO. Global dengue surveillance https://worldhealthorg.shinyapps.io/dengue_global/ Accessed Feb 19 2026

[2] Kraemer MUG, Sinka ME, Duda KA, Mylne AQN, Shearer FM, Barker CM, et al. The global distribution of the arbovirus vectors *Aedes aegypti *and *Ae. albopictus.* eLife. 2015;4:e08347. 10.7554/eLife.08347. 26126267 10.7554/eLife.08347 PMC4493616

[3] Kolimenakis A, Heinz S, Wilson ML, Winkler V, Yakob L, Michaelakis A, et al. The role of urbanisation in the spread of *Aedes* mosquitoes and the diseases they transmit-A systematic review. PLOS Negl Trop Dis. 2021;15:e0009631. 10.1371/journal.pntd.0009631. 34499653 10.1371/journal.pntd.0009631 PMC8428665

[4] Sugeno M, Kawazu EC, Kim H, Banouvong V, Pehlivan N, Gilfillan D, et al. Association between environmental factors and dengue incidence in Lao People’s Democratic Republic: A nationwide time-series study. BMC Public Health. 2023;23:2348. 10.1186/s12889-023-17277-0. 38012549 10.1186/s12889-023-17277-0 PMC10683213

[5] Zafar S, Overgaard HJ, Pongvongsa T, Vannavong N, Phommachanh S, Shipin O, et al. Epidemiological profile of dengue in Champasak and Savannakhet provinces, Lao People’s Democratic Republic, 2003–2020. Western Pac Surveill Response J. 2022;13:1–13. 10.5365/wpsar.2022.13.4.932. 36817500 10.5365/wpsar.2022.13.4.932 PMC9912291

[6] Ryan SJ, Carlson CJ, Mordecai EA, Johnson LR. Global expansion and redistribution of *Aedes*-borne virus transmission risk with climate change. PLOS Negl Trop Dis. 2019;13:e0007213. 10.1371/journal.pntd.0007213. 30921321 10.1371/journal.pntd.0007213 PMC6438455

[7] Nik Abdull Halim NMH, Che Dom N, Dapari R, Salim H, Precha NA. Systematic review and meta-analysis of the effects of temperature on the development and survival of the *Aedes* mosquito. Front Public Health. 2022;10:1074028. 10.3389/fpubh.2022.1074028. 36600940 10.3389/fpubh.2022.1074028 PMC9806355

[8] Liu Z, Zhang Q, Li L, He J, Guo J, Wang Z, et al. The effect of temperature on dengue virus transmission by Aedes mosquitoes. Front Cell Infect Microbiol. 2023;13:1242173. 10.3389/fcimb.2023.1242173. 37808907 10.3389/fcimb.2023.1242173 PMC10552155

[9] Morin CW, Comrie AC, Ernst K. Climate and dengue transmission: Evidence and implications. Environ Health Perspect. 2013;121:1264–72. 10.1289/ehp.1306556. 24058050 10.1289/ehp.1306556 PMC3855512

[10] Naish S, Dale P, Mackenzie JS, McBride J, Mengersen K, Tong S. Climate change and dengue: A critical and systematic review of quantitative modelling approaches. BMC Infect Dis. 2014;14:167. 10.1186/1471-2334-14-167. 24669859 10.1186/1471-2334-14-167 PMC3986908

[11] Polrob W, La-Up A. Nonlinear and lagged effects of climate variability on dengue incidence in an urban megacity: A distributed lag non-linear model (DLNM) based study in Bangkok. Thailand BMC Public Health. 2025;25:4024. 10.1186/s12889-025-25420-2. 41254624 10.1186/s12889-025-25420-2 PMC12625335

[12] Lambrechts L, Paaijmans KP, Fansiri T, Carrington LB, Kramer LD, Thomas MB, et al. Impact of daily temperature fluctuations on dengue virus transmission by *Aedes aegypti*. Proc Natl Acad Sci U S A. 2011;108:7460–5. 10.1073/pnas.1101377108. 21502510 10.1073/pnas.1101377108 PMC3088608

[13] Liyanage P, Tissera H, Sewe M, Quam M, Amarasinghe A, Palihawadana P, et al. A spatial hierarchical analysis of the temporal influences of the El Niño-southern oscillation and weather on dengue in Kalutara District, Sri Lanka. Int J Environ Res Public Health. 2016;13:1087. 10.3390/ijerph13111087. 27827943 10.3390/ijerph13111087 PMC5129297

[14] Thirumalai K, DiNezio PN, Okumura Y, Deser C. Extreme temperatures in Southeast Asia caused by el Niño and worsened by global warming. Nat Commun. 2017;8:15531. 10.1038/ncomms15531. 28585927 10.1038/ncomms15531 PMC5467164

[15] Eggeling J, Gao C, An D, Cruz-Cano R, He H, Zhang L, et al. Spatiotemporal link between el Niño Southern Oscillation (ENSO), extreme heat, and thermal stress in the Asia-Pacific region. Sci Rep. 2024;14:7448. 10.1038/s41598-024-58288-0. 38548842 10.1038/s41598-024-58288-0 PMC10978954

[16] Lin S, Dong B, Yang S. Enhanced impacts of ENSO on the Southeast Asian summer monsoon under global warming and associated mechanisms. Geophys Res Lett. 2024. 10.1029/2023GL106437.

[17] National Weather Service. Climate prediction Center—ONI https://www.cpc.ncep.noaa.gov/products/analysis_monitoring/ensostuff/ONI_v5.php Accessed 21 Feb 2026

[18] NOAA. DMI: Physical sciences laboratory https://psl.noaa.gov/data/timeseries/month/DMI/ Accessed 21 Feb 2026

[19] Chen Y, Xu Y, Wang L, Liang Y, Li N, Lourenço J, et al. Indian Ocean temperature anomalies predict long-term global dengue trends. Science. 2024;384:639–46. 10.1126/science.adj4427. 38723095 10.1126/science.adj4427

[20] Feng F, Ma Y, Qin P, Zhao Y, Liu Z, Wang W, et al. Temperature-driven dengue transmission in a changing climate: Patterns, trends, and future projections. GeoHealth. 2024. 10.1029/2024GH001059. 39347019 10.1029/2024GH001059 PMC11436633

[21] Tsai P-J, Lin T-H, Teng H-J, Yeh H-C. Critical low temperature for the survival of *Aedes aegypti* in Taiwan. Parasit Vectors. 2018;11:22. 10.1186/s13071-017-2606-6. 29310716 10.1186/s13071-017-2606-6 PMC5759216

[22] Chen W-J. Dengue outbreaks and the geographic distribution of dengue vectors in Taiwan: a 20-year epidemiological analysis. Biomed J. 2018;41:283–9. 10.1016/j.bj.2018.06.002. 30580791 10.1016/j.bj.2018.06.002 PMC6306330

[23] Thomas SM, Obermayr U, Fischer D, Kreyling J, Beierkuhnlein C. Low-temperature threshold for egg survival of a post-diapause and non-diapause European aedine strain, *Aedes albopictus* (Diptera: Culicidae). Parasit Vectors. 2012;5:100. 10.1186/1756-3305-5-100. 22621367 10.1186/1756-3305-5-100 PMC3403971

[24] Tsunoda T, Cuong TC, Dong TD, Yen NT, Le NH, Phong TV, et al. Winter refuge for *Aedes aegypti *and *Ae. albopictus* mosquitoes in Hanoi during Winter. PLOS One. 2014;9:e95606. 10.1371/journal.pone.0095606. 24752230 10.1371/journal.pone.0095606 PMC3994068

[25] Soukavong M, Thinkhamrop K, Pratumchart K, Soulaphy C, Xangsayarath P, Mayxay M, et al. Bayesian spatio-temporal analysis of dengue transmission in Lao PDR. Sci Rep. 2024;14:21327. 10.1038/s41598-024-71807-3. 39266587 10.1038/s41598-024-71807-3 PMC11393087

[26] Zafar S, Rocklöv J, Paul RE, Shipin O, Rahman MdS, Pientong C, et al. Landscape and climatic drivers of dengue fever in Lao People’s Democratic Republic and Thailand: A retrospective analysis during 2002–2019. Landsc Ecol. 2025;40:102. 10.1007/s10980-025-02102-3.

[27] Calvez E, Pommelet V, Somlor S, Pompon J, Viengphouthong S, Bounmany P, et al. Trends of the dengue Serotype-4 circulation with epidemiological, phylogenetic, and entomological insights in Lao PDR between 2015 and 2019. Pathogens. 2020;9:728. 10.3390/pathogens9090728. 32899416 10.3390/pathogens9090728 PMC7557816

[28] Troupin C, Intavong K, Somlor S, Viengphouthong S, Keosenhom S, Chindavong TA, et al. Molecular epidemiology of dengue viruses in Lao People’s Democratic Republic, 2020–2023. Microorganisms. 2025;13:318. 10.3390/microorganisms13020318. 40005687 10.3390/microorganisms13020318 PMC11857872

[29] Phanhkongsy S, Suwannatrai A, Thinkhamrop K, Somlor S, Sorsavanh T, Tavinyan V, et al. Spatial analysis of dengue fever incidence and serotype distribution in Vientiane Capital, Laos: a multi-year study. Acta Trop. 2024;256:107229. 10.1016/j.actatropica.2024.107229. 38768698 10.1016/j.actatropica.2024.107229

[30] Togami E, Chiew M, Lowbridge C, Biaukula V, Bell L, Yajima A, et al. Epidemiology of dengue reported in the World Health Organization’s western Pacific Region, 2013–2019. Western Pac Surveill Response J. 2023;14:1–16. 10.5365/wpsar.2023.14.1.973. 37064541 10.5365/wpsar.2023.14.1.973 PMC10090032

[31] Gasparrini A, Armstrong B, Kenward MG. Distributed lag non-linear models. Stat Med. 2010;29:2224–34. 10.1002/sim.3940. 20812303 10.1002/sim.3940 PMC2998707

[32] Gasparrini A. Distributed lag linear and non-linear models in R: the package dlnm. J Stat Softw. 2021;43:1–20. PMC3191524 22003319

[33] Yang X, Bao Y, Song Z, Shu Q, Song Y, Wang X, et al 2023 Key to ENSO phase-locking simulation: Effects of sea surface temperature diurnal amplitude. npj Clim Atmos Sci 6 159 10.1038/s41612-023-00483-3

[34] Lee S-K, Mapes BE, Wang C, Enfield DB, Weaver SJ. Springtime ENSO phase evolution and its relation to rainfall in the continental U.S. Geophys Res Lett. 2014;41:1673–80. 10.1002/2013GL059137.

[35] Meyers G, McIntosh P, Pigot L, Pook M. The years of El Niño, La Niña, and interactions with the tropical Indian Ocean. J Clim. 2007;20:2872–80. 10.1175/JCLI4152.1.

[36] Almahri AB, Hasanean HM, Labban AH. Teleconnections between the Pacific and Indian Ocean SSTs and the tropical cyclone activity over the Arabian Sea. Climate. 2025;13:193. 10.3390/cli13090193.

