# Supplementary material for: Climate drivers and winter constraints of dengue epidemics: a 10-year epidemiological perspective study in the Lao People’s Democratic Republic
Source: Infect Dis Poverty. 2026 May 1;15:46. doi: 10.1186/s40249-026-01438-5 (PMC13134311; doi:10.1186/s40249-026-01438-5)
Supplement: Supplementary file 1 — Supplementary Material 1. [file 40249_2026_1438_MOESM1_ESM.docx]

Supplementary materials

Table S1. A regional reclassification of provinces in Lao PDR

| Regional classification | The Province in Lao PDR |
| --- | --- |
| Northern Mountains and Highlands | Phongsaly, Luangnamtha, Oudomxay, Bokeo, Luangprabang, Sayabouly |
| Northeastern Highlands | Huaphanh, Xiengkhouang |
| Capital Region | Vientiane capital |
| Central Plains and River Basins | Vientiane province, Bolikhamxay, Khammuan, Saysomboun |
| Southern Mekong River Basin/Plains | Savannakhet, Champasak |
| Southern Mountains and Tropical Rainforests | Saravanh, Sekong, Attapeu |

Table S2. Summary of datasets and their sources

| Data | Source |
| --- | --- |
| Dengue cases data in Lao PDR (2014-2023) | National Center for Laboratory and Epidemiology, Ministry of Health |
| Provincial-level population statistics | Lao Statistics Bureau  (https://laosis.lsb.gov.la/main.do) |
| Local meteorological data (temperature and precipitation) in Lao PDR (2014-2023) | Lao Statistics Bureau  (https://laosis.lsb.gov.la/main.do) |

Table S3. Sensitivity analysis for model selection

| Model | Lag (months) | Sum of QAIC for 6 regions | △QAIC | Mean adjusted  Pseudo-R^2^ (Range) |
| --- | --- | --- | --- | --- |
| Main model | 3 | 666.24 |  | 0.78 (0.59-0.85) |
| 1 | 0 | 604.76 | -61.48 | 0.76 (0.59-0.85) |
| 2 | 1 | 627.39 | -38.85 | 0.76 (0.58-0.85) |
| 3 | 2 | 651.40 | -14.84 | 0.77 (0.59-0.85) |
| 4 | 4 | 688.44 | +22.20 | 0.78 (0.58-0.86) |
| 5 | 5 | 702.93 | +36.69 | 0.79 (0.58-0.87) |
| 6 | 6 | 716.86 | +50.62 | 0.78 (0.57-0.87) |
| 7 | 7 | 742.58 | +76.34 | 0.79 (0.56-0.87) |
| 8 | 8 | 768.02 | +101.78 | 0.79 (0.58-0.88) |

Table S4. Region-specific and nationwide cumulative relative risks (RRs) with 95% confidence intervals across ONI

| Regional classification | ONI | | | | | | |
| --- | --- | --- | --- | --- | --- | --- | --- |
|  | -1.0 | -0.5 | 0.0 | 0.5 | 1.0 | 1.5 | 2.0 |
| Northern Mountains and Highlands | 0.53  (0.36–0.78) | 0.73  (0.60–0.88) | 1.00  (0.99–1.01) | 1.38  (1.13–1.67) | 1.89  (1.28–2.78) | 2.60  (1.45–4.63) | 3.57  (1.65–7.72) |
| Northeastern Highlands | 0.94  (0.39–2.27) | 0.97  (0.62–1.51) | 1.00  (0.97–1.03) | 1.03  (0.66–1.60) | 1.06  (0.44–2.57) | 1.10  (0.29–4.12) | 1.13  (0.19–6.61) |
| Capital Region | 0.78  (0.45–1.34) | 0.88  (0.67–1.16) | 1.00  (0.98–1.02) | 1.14  (0.87–1.49) | 1.29  (0.75–2.22) | 1.46  (0.65–3.31) | 1.66  (0.56–4.94) |
| Central Plains and River Basins | 0.41  (0.31–0.53) | 0.64  (0.56–0.73) | 1.00  (0.99–1.01) | 1.57  (1.37–1.80) | 2.45  (1.87–3.22) | 3.84  (2.56–5.77) | 6.02  (3.51–10.34) |
| Southern Mekong River Basin/Plains | 0.38  (0.27–0.55) | 0.62  (0.52–0.74) | 1.00  (0.99–1.01) | 1.62  (1.35–1.95) | 2.63  (1.83–3.79) | 4.27  (2.48–7.36) | 6.93  (3.36–14.30) |
| Southern Mountains and Tropical Rainforests | 0.97  (0.74–1.26) | 0.98  (0.86–1.12) | 1.00  (0.99–1.01) | 1.02  (0.89–1.16) | 1.03  (0.79–1.35) | 1.05  (0.71–1.56) | 1.07  (0.63–1.81) |

Table S5. Region-specific and nationwide cumulative relative risks (RRs) with 95% confidence intervals across DMI

| Regional classification | DMI | | | | | |
| --- | --- | --- | --- | --- | --- | --- |
|  | -1.0 | -0.5 | 0.0 | 0.5 | 1.0 | 1.5 |
| Northern Mountains and Highlands | 2.36  (1.39–4.03) | 1.54  (1.18–2.01) | 1.00  (0.98–1.02) | 0.65  (0.50–0.85) | 0.42  (0.25–0.72) | 0.28  (0.12–0.61) |
| Northeastern Highlands | 2.19  (0.75–6.41) | 1.48  (0.87–2.54) | 1.00  (0.96–1.04) | 0.68  (0.40–1.16) | 0.46  (0.16–1.34) | 0.31  (0.06–1.55) |
| Capital Region | 2.69  (1.41–5.13) | 1.64  (1.19–2.27) | 1.00  (0.98–1.03) | 0.61  (0.44–0.84) | 0.37  (0.20–0.71) | 0.23  (0.09–0.60) |
| Central Plains and River Basins | 1.38  (0.90–2.10) | 1.17  (0.95–1.45) | 1.00  (0.99–1.02) | 0.85  (0.69–1.05) | 0.73  (0.48–1.11) | 0.62  (0.33–1.17) |
| Southern Mekong River Basin/Plains | 2.07  (1.16–3.68) | 1.44  (1.08–1.92) | 1.00  (0.98–1.02) | 0.70  (0.52–0.93) | 0.48  (0.27–0.86) | 0.34  (0.14–0.80) |
| Southern Mountains and Tropical Rainforests | 1.44  (0.97–2.13) | 1.20  (0.99–1.46) | 1.00  (0.99–1.01) | 0.84  (0.69–1.02) | 0.70  (0.47–1.03) | 0.58  (0.32–1.05) |


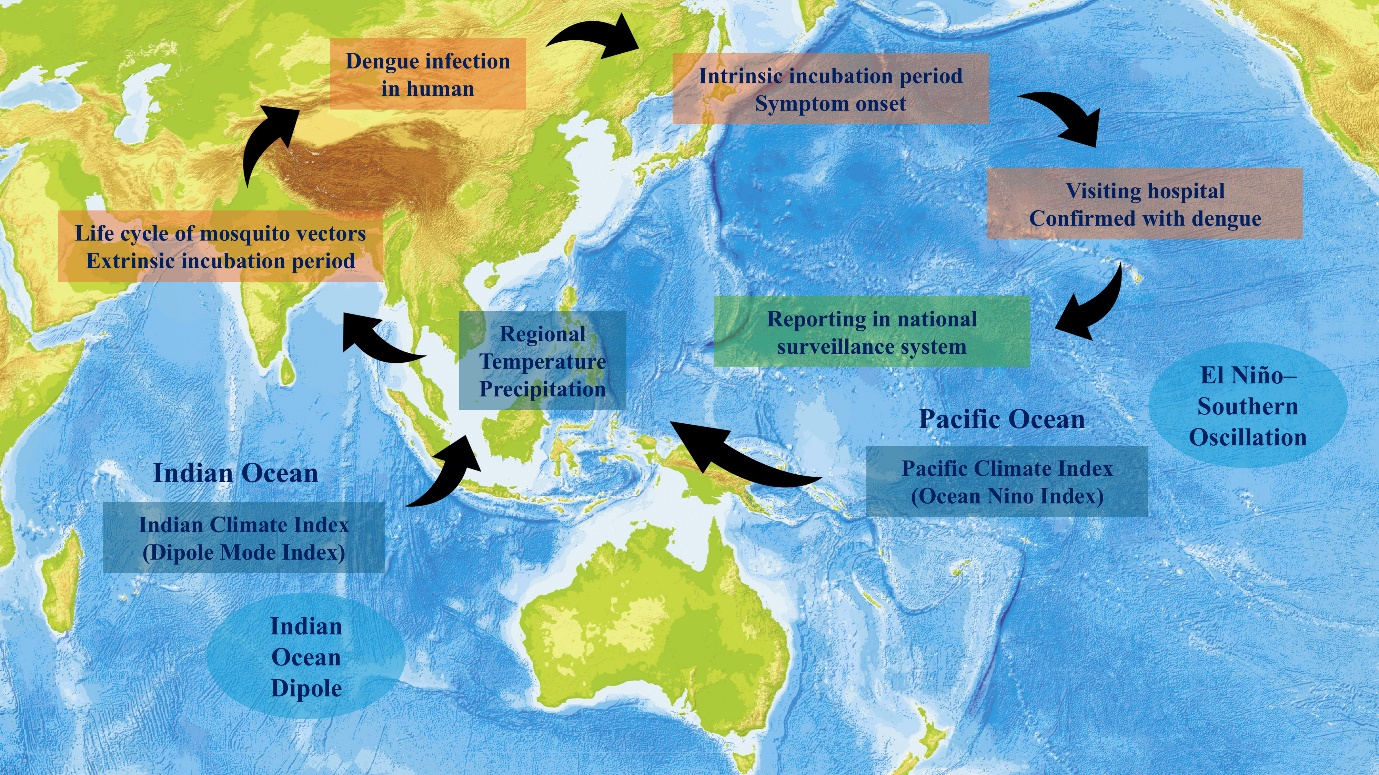


Figure S1. Conceptual framework linking large-scale oceanic climate variability to dengue transmission and its surveillance


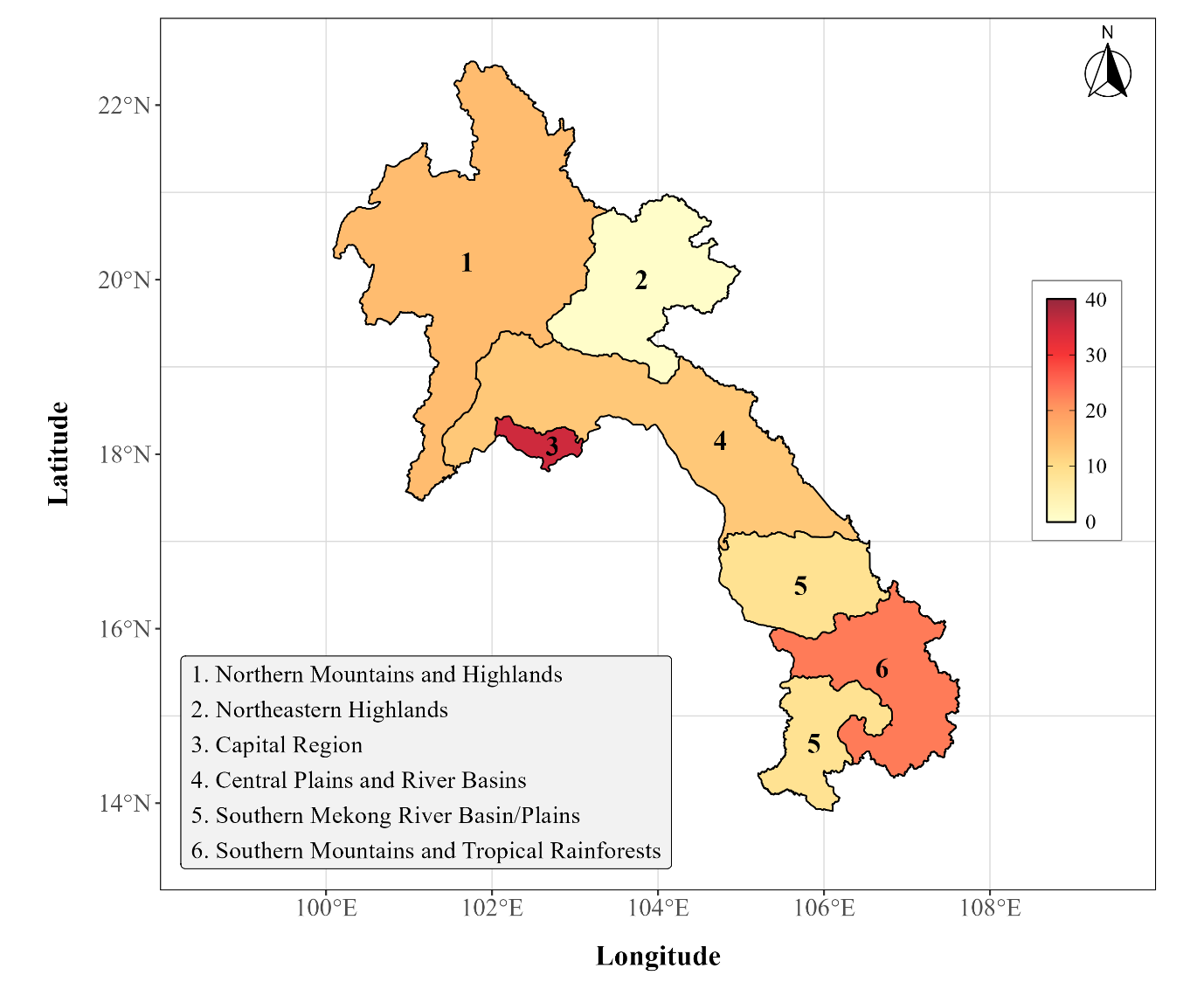


Figure S2. Average dengue incidence (per 100,000) by region in Lao PDR, 2014-2023


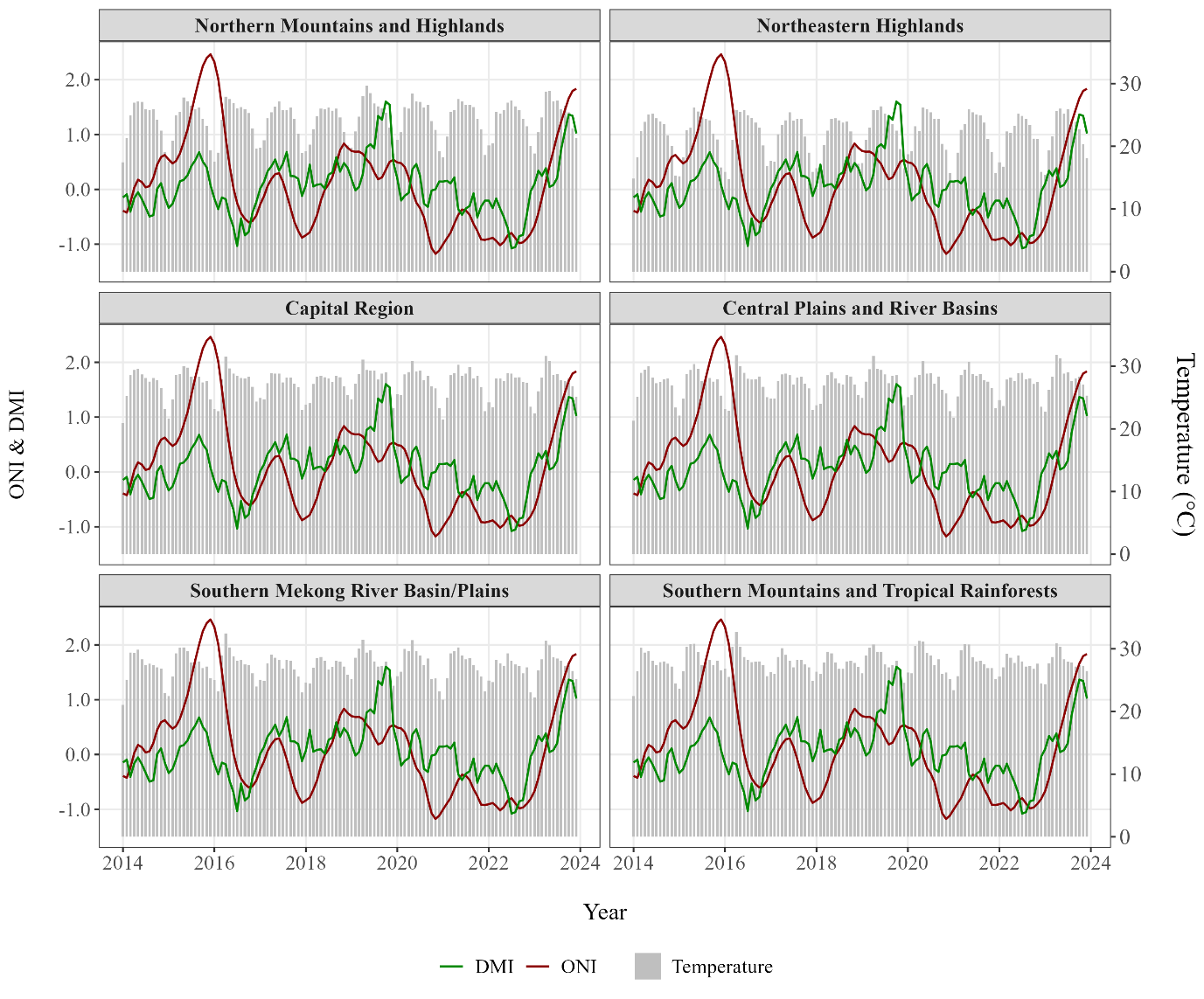


Figure S3. Climate indices (ONI, DMI) and average temperatures by region in Lao PDR


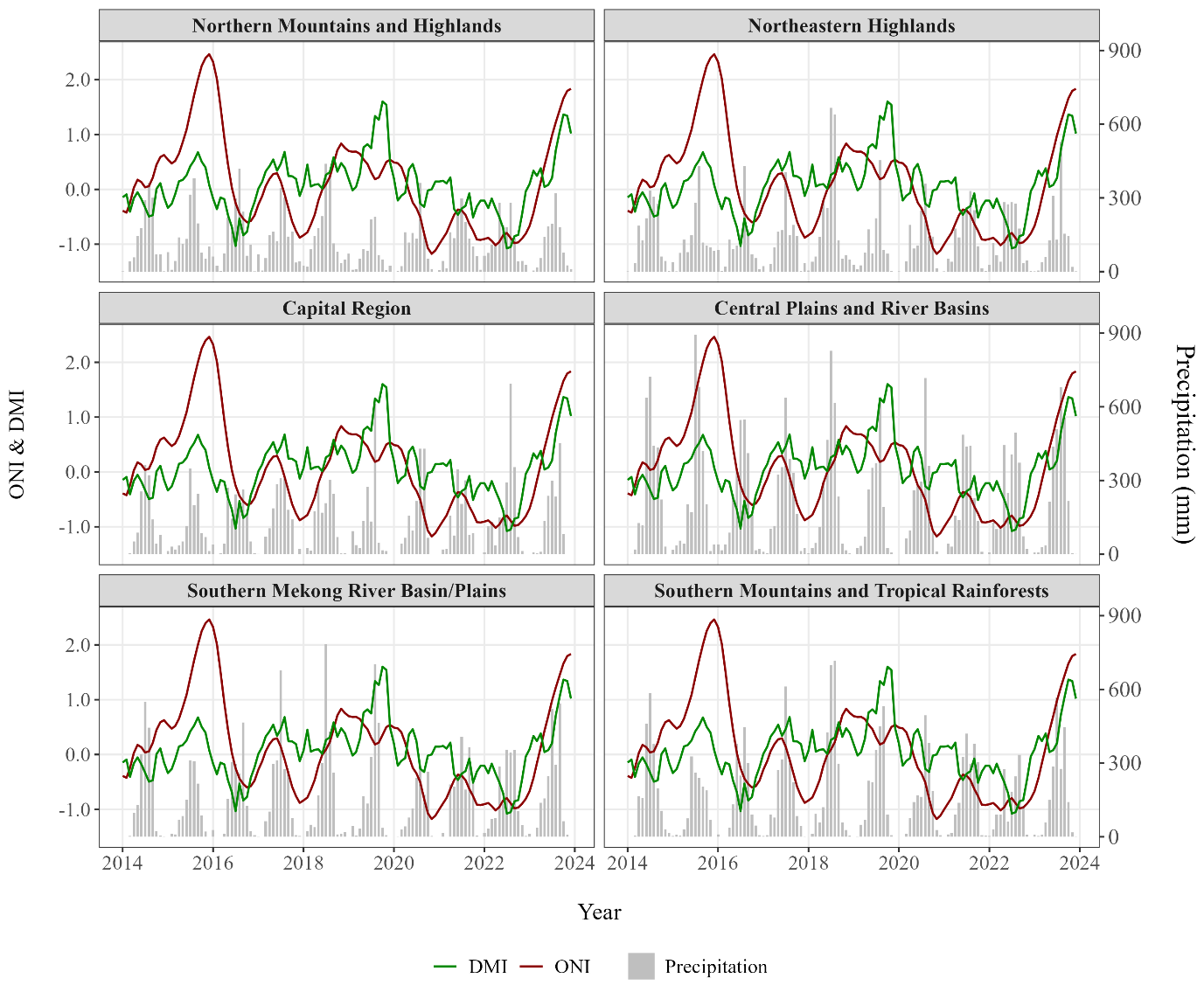


Figure S4. Climate indices (ONI, DMI) and Precipitation by region in Lao PDR


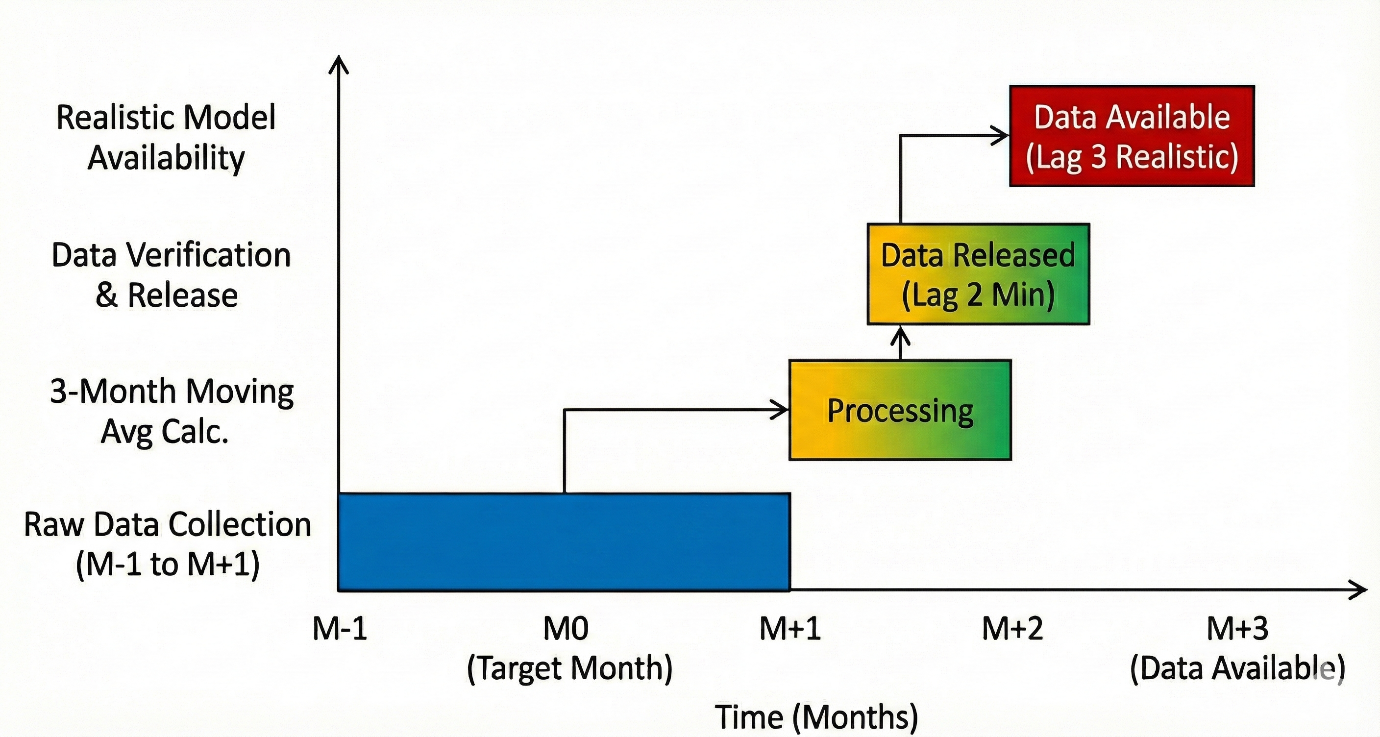


Figure S5. Operational feasibility considering ONI/DMI data availability and biological plausibility in real world


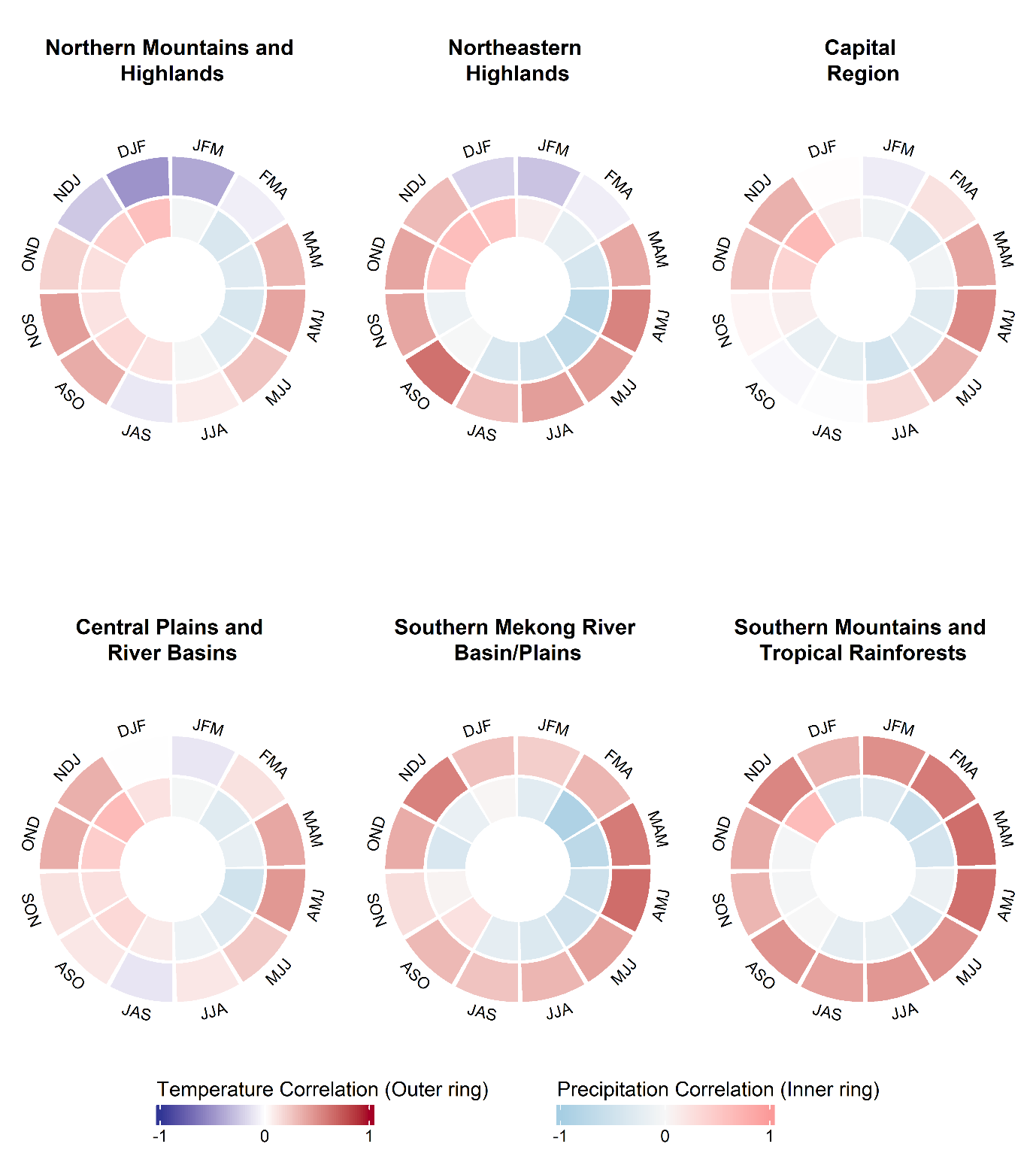


Figure S6. Correlation between ONI and monthly temperature and precipitation in each region


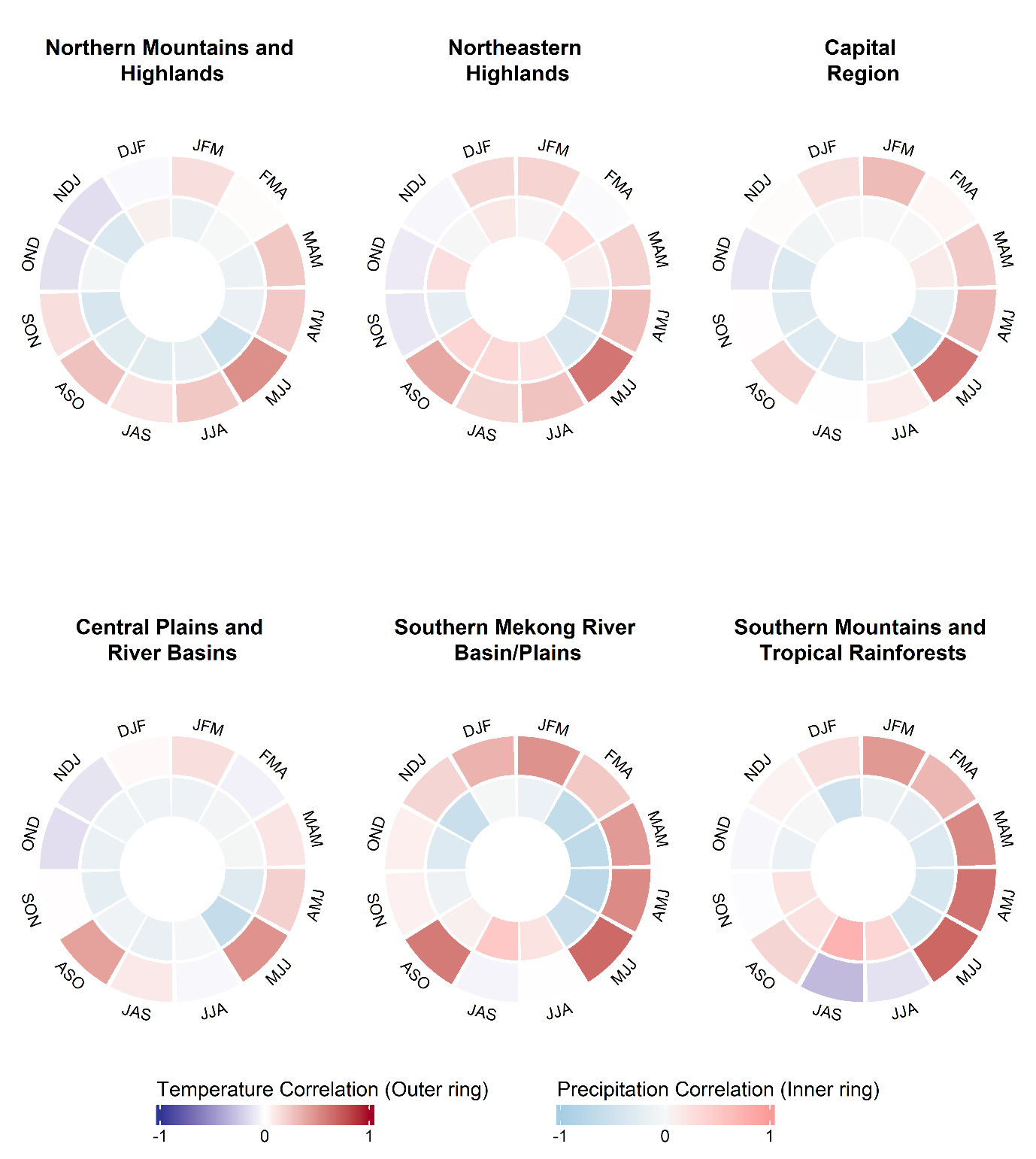


Figure S7. Correlation between DMI and monthly temperature and precipitation in each region
